# Exposure to di(2-ethylhexyl) phthalate inhibits luteal function via dysregulation of CD31 and prostaglandin F2alpha in pregnant mice

**DOI:** 10.1186/s12958-015-0013-4

**Published:** 2015-03-03

**Authors:** Meijun Guo, Lidan Lai, Teng Zong, Yan Lin, Bei Yang, Lu Zhang, Mo Li, Haibin Kuang

**Affiliations:** Department of Physiology, School of Medicine, Nanchang University, Nanchang, Jiangxi China; Department of Obstetrics and Gynecology, Hospital of Jixi Province People, Nanchang, Jiangxi China

**Keywords:** DEHP, Corpora lutea, Pregnancy, Progesterone

## Abstract

**Background:**

Di(2-ethylhexyl) phthalate (DEHP) exposure reduces embryo implantations, increases embryonic loss, and decreases fetal body weights. However, whether it is associated with the alteration of luteal function remains unknown. Thus, our aim in this study was to explore the effect and mechanism of DEHP on luteal function in pregnant mice in vivo.

**Methods:**

Mice were administered DEHP by gavage at 125, 250, 500 mg/kg/day from gestational days (GD) 1 to 9 or 13. Levels of serum progesterone and estradiol were measured by radioimmunoassay. The numbers and sizes of corpora lutea were calculated by ovarian histomorphology. Steroidogenic enzymes were assessed by qRT-PCR. CD31 protein was detected by immunocytochemistry, and prostaglandin F2alpha (PGF2alpha) levels were evaluated by enzyme immunoassay.

**Results:**

Treatment with DEHP significantly inhibited progesterone secretion in pregnant mice in a dose-dependent manner but did not inhibit estradiol production on GD 9 and 13. Treatment also showed concomitant decreases in transcript levels for key steroidogenic enzymes (CYP11A, 3β-HSD, and StAR) on GD 13. Furthermore, DEHP administration significantly reduced the numbers and sizes of corpora lutea on GD 13. No significant changes in the ratio of ovary weight vs. body weight were observed between the control group and treated animals on GD 9 and 13. In addition, treatment with DEHP significantly inhibited CD31 expression of corpora lutea, whereas plasma PGF2alpha levels in DEHP treatment groups were significantly higher compared with the control groups on GD 9 and 13.

**Conclusions:**

The results show DEHP significantly inhibits luteal function of pregnant mice in vivo, with a mechanism that seems to involve the down-regulation of progesterone and steroidogenic enzymes message RNA, the decrease in CD31 expression, and the increase in PGF2alpha secretion.

**Electronic supplementary material:**

The online version of this article (doi:10.1186/s12958-015-0013-4) contains supplementary material, which is available to authorized users.

## Background

Di(2-ethylhexyl) phthalate (DEHP) is used as a plasticizer in flexible polyvinyl chloride (PVC) products, including food and beverage packaging, cosmetics, medical devices, construction products and children’s toys [1]. Currently, DEHP is the most widespread phthalate, accounting for approximately 50% of the market for PVC plasticizers in the European Union [2,3]. DEHP loosely binds to plastic materials, so it easily leaches out of these products and enters into the environment over time and with product use. The Agency for Toxic Substances and Disease Registry estimates that the maximum daily exposure to DEHP for the general population is approximately 2 mg/day [4]. However, occupational, medical and dust exposures can lead to much higher levels of exposure. For example, exposure to DEHP from blood transfusions can be as high as 250–300 mg, equivalent to a dose of 3.5-4.3 mg/kg for an adult weighing 70 kg, and the levels of DEHP in household dust can reach as high as 400–700 mg/kg [4,5].

Recently, emerging evidence has suggested that exposure to DEHP has potential hazardous effects on animal and human health, such as developmental toxicity, immunotoxicity, neurotoxicity and especially reproductive toxicity [4]. In males, studies showed that exposure to DEHP increased germ cell apoptosis, disrupted spermatogenesis and inhibited steroidogenesis of Leydig cells [6-9]. Women are exposed to DEHP more frequently than men due to its widespread use in nail polish, shampoos and cosmetics [10]. Studies in vivo demonstrated that DEHP exposure prolonged estrous cycles, decreased serum estradiol levels and inhibited ovulations in adult rats. In vitro, mono-(2-ethylhexyl) phthalate (MEHP, the active metabolite of DEHP) decreases granulosa cell aromatase message RNA and protein levels [4,11]. Chronic and long-term occupational exposure to phthalates has been associated with endometriosis, lower rates of pregnancy, high miscarriage rates and pregnancy complications such as anemia and hypertensive disorders in women [12,13]. In female laboratory rats, DEHP reduces endometrial receptivity and embryo implantations, increases embryonic loss, and decreases fetal body weights [4,14,15]. However, whether it is associated with the alteration of luteal function remains unknown. Thus, our aim in this study was to explore the effect and mechanism of DEHP on early- and mid-luteal function in pregnant mice. Our results indicated that daily exposure to DEHP inhibits luteal function in pregnant mice through a mechanism that seems to involve progesterone and steroidogenic enzymes down-regulation, a decrease in CD31 expression and an increase in PGF2alpha secretion.

## Methods

### Animals and treatments

Adult female CD-1 mice (25–30 g) were contained in a consistent photoperiod (14 h light: 10 h dark cycle) and allowed free access to food and water. The experimental protocols were approved by the Institutional Animal Care and Use Committee of Nanchang University (Permit Number: NJ20130616). The ethical approval date was June 16, 2013 and the study was initiated July, 2013. Adult virgin female mice were mated with fertile males of the same strain to induce pregnancy. The morning following the identification of a vaginal plug was designated as day 1 (D1) of gestation. The pregnant animals were divided into 4 groups as follows (n = 20/group): (1) control group, (2) 125 mg/kg/day DEHP group, (3) 250 mg/kg/day DEHP group and (4) 500 mg/kg/day DEHP group. From D1 until the conclusion of the experiment, pregnant female mice were weighed and administered DEHP (Guo Lei Chemicals, China) dissolved in sesame oil (Sigma, USA) at various concentrations (125, 250 and 500 mg/kg/day) by gavage (0.1 ml/10 g body weight). The control group mice were given the same volume of sesame oil. Treated mice were sacrificed for ovary collection between 15:00 and 16:00 on gestational day (GD) 9 and 13 by cervical dislocation (10 mice for each group on GD9 and GD13), respectively. Ovaries from these mice were frozen in liquid nitrogen for further analysis.

### Ovarian histomorphology

Tissues were fixed in Bouin’s solution for 12 h, dehydrated in ethanol, and embedded in paraffin. Tissue sections were cut at 5 μm and stained with hematoxylin and eosin for morphological evaluation. The numbers of corpora lutea were calculated by counting the numbers in both ovaries and generating an average number per individual. Images were captured in six ovary sections per animal using digital camera head DS-Fi1 (Nikon, Japan), and the sizes of the corpora lutea were measured using ImageJ (v. 1.45 s, NIH).

### Immunohistochemistry

Tissue sections (5 μm) were deparaffinized in xylene and hydrated in graded ethanol solutions followed by water. Sections were placed in 0.01 M citric acid (pH = 6) and microwaved for 15 min to expose the antigens. Endogenous peroxidase activity was blocked by incubating the sections in 3% hydrogen peroxide in PBS for 10 min. Nonspecific binding was blocked in 5% BSA in PBS for 60 min. Then, the sections were incubated with rabbit anti-CD31 (1:150, Santa Cruz, USA) overnight at 4°C. After washing in PBS, the sections were incubated with a secondary antibody for 45 min at 37°C. The primary antibody was detected with fresh diaminobenzidine solution, in conjunction with counter-staining using Harris’ hematoxylin. In some sections, the primary antibodies were replaced with rabbit preimmune IgG as a negative control.

### Quantitative PCR

Total RNA was extracted from ovary tissues with RNAiso Plus solution (TaKaRa, China) according to the manufacturer’s protocol. RNA samples were reverse-transcribed into single-stranded cDNA in a 25 μl reaction mixture (TaKaRa, China). Real-time PCR was then performed in a 20 μl reaction volume containing 10 μl of 2x Brilliant SYBR Green Mix (TaKaRa, China), 2 μl of template cDNA, 0.5 μM primers, and 300 nM reference dye using the ABI thermal cycler 7500. The thermal cycling conditions were 95°C for 30 sec, followed by 40 cycles at 94°C for 5 sec and 60°C for 34 sec. Melting curve analysis and agarose gel electrophoresis were conducted following the quantitative PCR assays to monitor PCR product purity. The results were analyzed using ABI Prism 7500 software (Applied Biosystems, USA), and18S identified to be stable in the DEHP-treated experiment was used as an internal control. The following primers were used: 18S (ACCESSION:NR_003278): sense, 5′-AAT CAG GGT TCG ATT CCG GA--3′; antisense, 5′-CCA AGA TCC AAC TAC GAG CT-3′. StAR (ACCESSION:NM_011485): sense, 5′-CGC AGA GGT TCC ACC TGT GT-3′; antisense, 5′-TCC GGC ATC TCC CCA AA-3. CYP11A(ACCESSION:NM_019779): sense, 5′-CCG GAG CGG TTC CTT GT-3′; antisense, 5′-CCA ATG GGC CTC TGA TAA TAC TG-3′. 3β-HSD(ACCESSION:NM_013821): sense, 5′-GGA GGA AGC CAA GCA GAA AA-3′; antisense, 5′-CCC TGT GCT GCT CCA CTA GTG-3′.

### Enzyme immunoassay (EIA)

Concentrations of PGF2alpha were measured in plasma using a commercially available EIA kit (Cayman Chemical) according to the manufacturer’s instructions. The intra- and inter-assay coefficients of variation did not exceed 7.1% and 8.2%.

### Hormone measurements

The plasma concentrations of estradiol (E2) and progesterone (P4) were measured using specific RIA kits (Jiuding Medicine Biotechnology Co, China). Samples were detected in duplicate. The intra- and inter-assay coefficients of variation using these kits did not exceed 10%. The cross-reactivities with other peptides and steroid hormones in these kits did not exceed 4%. The detection limits of the E2 and P4 kits are 1 pg/ml and 0.25 ng/ml, respectively.

### Statistical analysis

The data are presented as the means +/− SD. The results were analyzed by using one-way ANOVA followed by LSD’s post-hoc test. A value of P < 0.05 was considered to be statistically significant. All statistical analyses were performed using the Statistical Package for the Social Sciences (SPSS, Chicago, IL) 13.0.

## Results

### DEHP administration inhibits luteal function in pregnant mice

**Figure 1:**
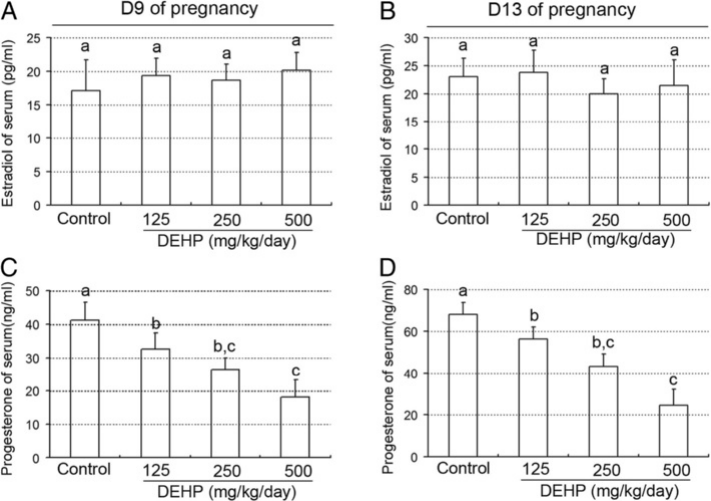
**Effect of DEHP exposure on serum estradiol and progesterone levels. (A)** Serum estradiol levels on day 9 of pregnancy. **(B)** Serum estradiol levels on day 13 of pregnancy. **(C)** Serum progesterone levels on day 9 of pregnancy. **(D)** Serum progesterone levels on day 13 of pregnancy. The results are shown as the means +/− SD of 10 animals. Groups with different superscript letters are significantly different (P < 0.05, ANOVA followed by LSD multiple range test).

**Figure 2:**
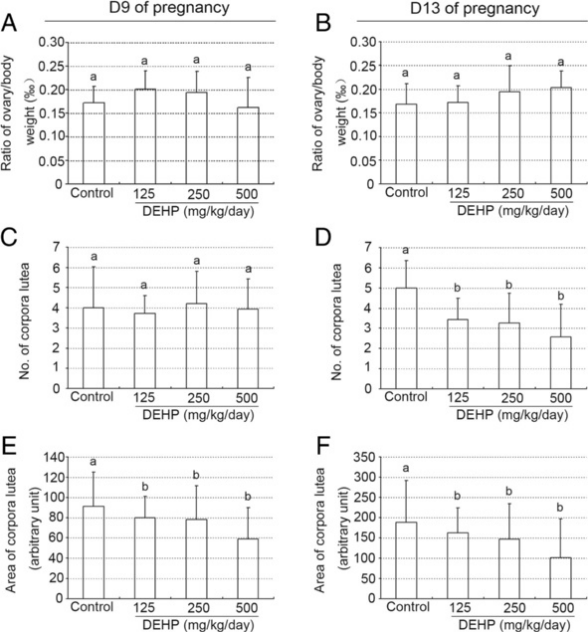
**Effect of DEHP exposure on the ovarian weights, numbers and sizes of corpora lutea. (A)** Ratio of ovary/body weight on day 9 of pregnancy. **(B)** Ratio of ovary/body weight on day 13 of pregnancy. **(C)** Numbers of corpora lutea on day 9 of pregnancy. **(D)** Numbers of corpora lutea on day 13 of pregnancy. **(E)** Sizes of corpora lutea on day 9 of pregnancy. **(F)** Sizes of corpora lutea on day 13 of pregnancy. The results are shown as the means +/− SD of 10 animals. Groups with different superscript letters are significantly different (P < 0.05, ANOVA followed by LSD multiple range test).

**Figure 3:**
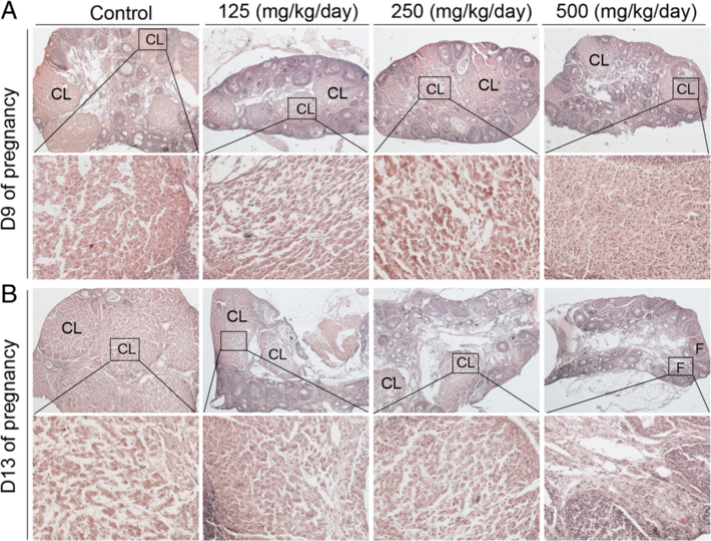
**Ovarian histomorphology detected by H&E staining. (A)** Representative images of ovary on day 9 of pregnancy. **(B)** Representative images of ovaries on day 13 of pregnancy. Squared areas at the top (×40) are presented at higher magnification (×200) at the bottom. CL, corpus luteum; F, follicle.

As shown in Figure 1A and B, treatment with DEHP did not affect estradiol levels, whereas it induced a significant decrease in plasma progesterone levels in a dose-dependent manner compared with the control group on GD 9 and 13 (all P < 0.01, Figure 1C and D). In addition, administration of DEHP did not affect the ratios of ovary weight to body weight on GD 9 and 13 (Figure 2A and B). No statistically significant difference related to the numbers of corpora lutea was observed between the control group and treated animals at any dose level on GD 9 (Figures 2C and 3A). However, the administration of various doses of DEHP significantly reduced the numbers of corpora lutea on GD13 (all P < 0.01, Figures 2D and 3B) and the sizes of corpora lutea on GD9 and 13 (P < 0.05 or 0.01, Figures 2E-F and 3B), compared to the control group.

### Effects of DEHP exposure on ovary steroidogenic enzymes in pregnant mice

**Figure 4:**
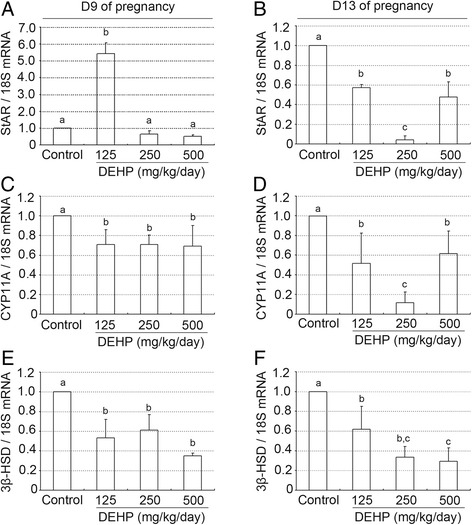
**Effect of DEHP exposure on the expression of different steroidogenic enzymes mRNA. (A, B)** StAR mRNA levels in the ovary on day 9 **(A)** and 13 **(B)** of pregnancy. **(C, D)** CYP11A mRNA levels in the ovary on day 9 **(C)** and 13 **(D)** of pregnancy. **(E, F)** 3β-HSD mRNA levels in the ovary on day 9 **(E)** and 13 **(F)** of pregnancy. mRNAs levels were quantified using quantitative PCR and were normalized to 18S. The data are represented as the means +/− SD of 10 animals. Groups with different superscript letters are significantly different (P < 0.05, ANOVA followed by LSD multiple range test).

To investigate the underlying mechanism of progesterone decrease from DEHP treatment, we detected the mRNA levels of three key steroidogenic enzymes: (1) steroidogenic acute regulatory protein (StAR) (Figure 4A,B), (2) cytochrome P45011A (CYP11A) (Figure 4C,D), and (3) 3-hydroxysteroid dehydrogenase (3β-HSD) (Figure 4E,F), by real-time PCR. Compared to the control group, administration of different doses of DEHP inhibited levels of CYP11A and 3β-HSD mRNA on GD 9 and 13 (Figure 4C-F). However, on gestational day 9, StAR mRNA levels were similar in the control and DEHP-treated animals (Figure 4A). Only the 125 mg DEHP/kg/day dose group showed significantly increased levels of StAR mRNA compared with the control group(P < 0.01, Figure 4A). In addition, treatment with DEHP significantly attenuated StAR mRNA levels compared to control on GD 13 (P < 0.05 or 0.01, Figure 4B).

### Effect of DEHP exposure on luteal vascularization and PGF2alpha levels in pregnant mice

**Figure 5:**
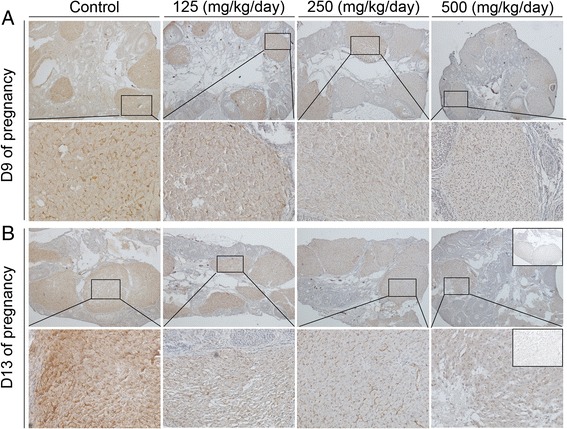
**Expression levels of CD31 protein detected by immunohistochemistry. (A, B)** Representative images of ovaries on day 9 **(A)** and 13 **(B)** of pregnancy. Squared areas at the top (×40) are presented at higher magnification (×200) at the bottom. Inset is negative control.

**Figure 6:**
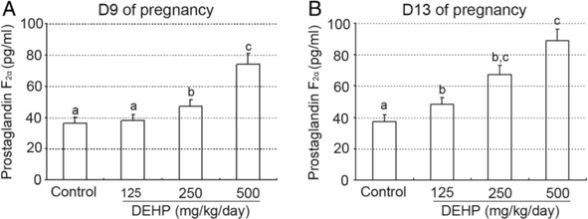
**Effect of DEHP exposure on prostaglandin F2alpha levels on day 9 and 13 of pregnancy. (A, B)** Plasma concentrations of PGF2alpha on day 9 **(A)** and 13 **(B)** of pregnancy. The results are shown as the means +/− SD of 10 animals. Groups with different superscript letters are significantly different (P < 0.05, ANOVA followed by LSD multiple range test).

As shown in Figure 5A and B, treatment with DEHP significantly inhibited vascularization of corpora lutea compared with the control group on GD 9 and 13 (Additional file 1), as evidenced through CD31 immunostaining. However, on gestational day 9, the 250 mg and 500 DEHP/kg/day dose groups showed significantly enhanced plasma PGF2alpha levels compared with the control group (P < 0.05, 0.01, Figure 6A). Moreover, plasma PGF2alpha levels in all DEHP treatment groups were significantly greater compared to the control group’s concentrations on GD 13 (P < 0.05 or 0.01, Figure 6B).

## Discussion

The present study is, to the best of our group’s knowledge, the first to examine the effects of DEHP on luteal function during mouse pregnancy in vivo. The results indicate that treatment with DEHP significantly inhibited progesterone secretion but did not inhibit estradiol production, with concomitant decreases in transcript levels for key steroidogenic enzymes (CYP11A, 3β-HSD, and StAR). Furthermore, DEHP administration significantly reduced the numbers and sizes of corpora lutea on gestational day 13 but did not reduce the ratios of ovary weights vs. body weighs on gestational days 9 and 13. In addition, administration of DEHP to pregnant mice was associated with the decrease in luteal CD31 expression and increase in serum PGF2alpha secretion. These findings suggest that increased miscarriages and decreased pregnancy rates in gestational mice exposed to DEHP are possibly mediated, at least in part, via suppression of ovary luteal function, a decrease in luteal CD31 expression and an increase in serum PGF2alpha secretion.

Progesterone plays a vital role in the maintenance of pregnancy and development of the fetus, and adequate levels of progesterone are essential for normal uterine decidualization and establishment of early pregnancy [16,17]. In the present study, we first explored the effects of DEHP on ovary luteal function during mouse pregnancy in vivo. The results showed that DEHP treatment significantly inhibited progesterone release in a dose-dependent manner but did not inhibit estradiol production. This is consistent with Francesco Parillo’s result [18], in which DEHP exposure also decreased plasma progesterone levels and corpora lutea production in early, mid, and late stages of rabbit pseudopregnancy. Furthermore, Federica Romani showed that DEHP and other phthalates negatively influence luteal steroidogenesis by reducing both basal and hCG-stimulated-progesterone release in highly purified human luteal cells [19]. In addition, Li et al. indicated that DEHP reduces progesterone levels and induces apoptosis of ovarian granulosa cells in adult female ICR mice [20]. However, Maria A. Herreros’s research [21] indicated that doses of 25 and 50 mg/kg of DEHP reduced the sizes of corpora lutea in sheep, but plasma progesterone levels in DEHP-treated ewes were unusually higher than in the control group. In addition, DEHP-exposed ewes had significantly higher plasma progesterone concentration from Day 2 of the luteal phase [22]. One possible explanation of this unexpected finding would be the fact that plasma progesterone concentrations in DEHP-treated sheep were elevated not as a result of an increased secretion but due to diminished clearance of progesterone. In fact, some studies showed that plasma progesterone levels were affected more by its metabolic clearance than by the level of secretion from corpora lutea [21]. The confirmation of this postulated mechanism requires further investigation.

Our data indicated that treatment with DEHP inhibited levels of CYP11A and 3β-HSD mRNA on gestational days 9 and 13 and inhibited levels of StAR mRNA on gestational day 13, while StAR mRNA levels were similar in control and DEHP-treated animals on gestational day 9. In vitro-cultured luteal cells, DEHP treatment diminished progesterone production and 3β-HSD protein levels in early and mid corpora lutea. However, in vivo DEHP treatment did not affect 3β-HSD protein in pseudopregnant rabbits [18]. In cultured murine leydig tumor cell line MLTC-1, CYP11A, CYP17, and 3β-HSD showed increased expression following exposure to DEHP, but some insignificant inhibitory effects appeared in the 10 μmol/L treatment group compared to the controls [7,23]. These results indicate that the effects of DEHP on the expression of steroidogenesis-related enzymes and progesterone production are species dependent, environment dependent and dose dependent.

PGF2alpha is recognized as having a decisive role in the functional regression of the corpus luteum [24]. Our results showed that treatment with DEHP significantly enhanced plasma PGF2alpha levels compared with the control group, suggesting that DEHP inhibited the luteal synthesis of progesterone by PGF2alpha-induced luteolysis in pregnant mice. In addition, Wang X et al. also indicated that DEHP stimulated secretion of PGF2alpha and inhibited secretion of PGE2 in cultured luteal cells from cows on days 8–12 of the estrous cycle [25]. Furthermore, our results indicated that DEHP treatment significantly induced the up-regulation of COX2 protein compared to control group (Additional files 2 and 3), which is key enzyme and catalyzes the rate-limiting step in prostaglandin biosynthesis. However, Romani F et al. demonstrated that PGF2alpha release and PGE2 release were both reduced after DEHP incubation in human luteal cells [19]. The data above suggest that ovarian luteolysis may be regulated by PGF2alpha release or by the dysregulation between luteotrophic and luteolytic factors, such as the ratios of PGF2alpha/PGE2. PGF2alpha decreases progesterone synthesis by a number of intracellular mechanisms, including downregulation of receptors for luteotropic hormones and decreased activity of the steroidogenic enzymes required for the biosynthesis of progesterone and the induction of cell death [24]. The precise mechanism of this effect requires further study.

Vascularization is essential for the development of a functional corpus luteum.

Our study has shown that treatment with DEHP significantly inhibited CD31 protein expression in the corpora lutea. Ban JB et al. showed that MEHP could induce apoptosis of human umbilical vein endothelial cells through a reactive oxygen species-mediated mitochondria-dependent pathway [26]. In addition, Federica Romani indicated that levels of VEGF protein in human luteal cells had a statistically significant decrease after incubation with DEHP [19]. Moreover, DEHP treatment negatively affects the development of retinal vessels in newborn rats [27]. These data suggest that DEHP negatively influences corpus luteum function by inhibiting luteal angiogenesis in pregnant mice.

## Conclusions

Exposure to DEHP significantly inhibited the luteal function of pregnant mice, with a mechanism that seems to involve the down-regulation of progesterone and steroidogenic enzymes, a decrease in CD31 expression and an increase in PGF2alpha secretion. Further studies are needed to identify the causes of these aberrations and their consequences to fertility. The systemic effect of DEHP treatment remains unknown in pregnant mice.

## Additional files

Additional file 1:
**Relative expression levels of CD31 protein detected by immunohistochemistry.** **(A, B)** Relative expression levels of CD31 protein in the ovaries on day 9 **(A)** and 13 **(B)** of pregnancy. Groups with different superscript letters are significantly different (P < 0.05, ANOVA followed by LSD multiple range test). Additional file 2:
**The expression of COX2 protein detected by immunohistochemistry.** **(A, B)** Representative images of ovaries on day 9 **(A)** and 13 **(B)** of pregnancy. Squared areas at the top (×100) are presented at higher magnification (×200) at the bottom. Inset is negative control. CL, corpus luteum. Additional file 3:
**Relative expression levels of COX2 protein detected by immunohistochemistry.** **(A, B)** Relative expression levels of COX2 protein in the ovaries on day 9 **(A)** and 13 **(B)** of pregnancy. Groups with different superscript letters are significantly different (P < 0.05, ANOVA followed by LSD multiple range test).

## Abbreviations

DEHP: Di(2-ethylhexyl) phthalate
EIA: Enzyme immunoassay
GD: Gestational days
MEHP: Mono-(2-ethylhexyl) phthalate
PGF2alpha: Prostaglandin F2alpha
PVC: Polyvinyl chloride
qRT-PCR: Quantitative reverse transcription PCR
