# Supplementary figures and images for: Exposure to di(2-ethylhexyl) phthalate inhibits luteal function via dysregulation of CD31 and prostaglandin F2alpha in pregnant mice
Source: Reprod Biol Endocrinol. 2015 Mar 3;13:11. doi: 10.1186/s12958-015-0013-4 (PMC4351920; doi:10.1186/s12958-015-0013-4)

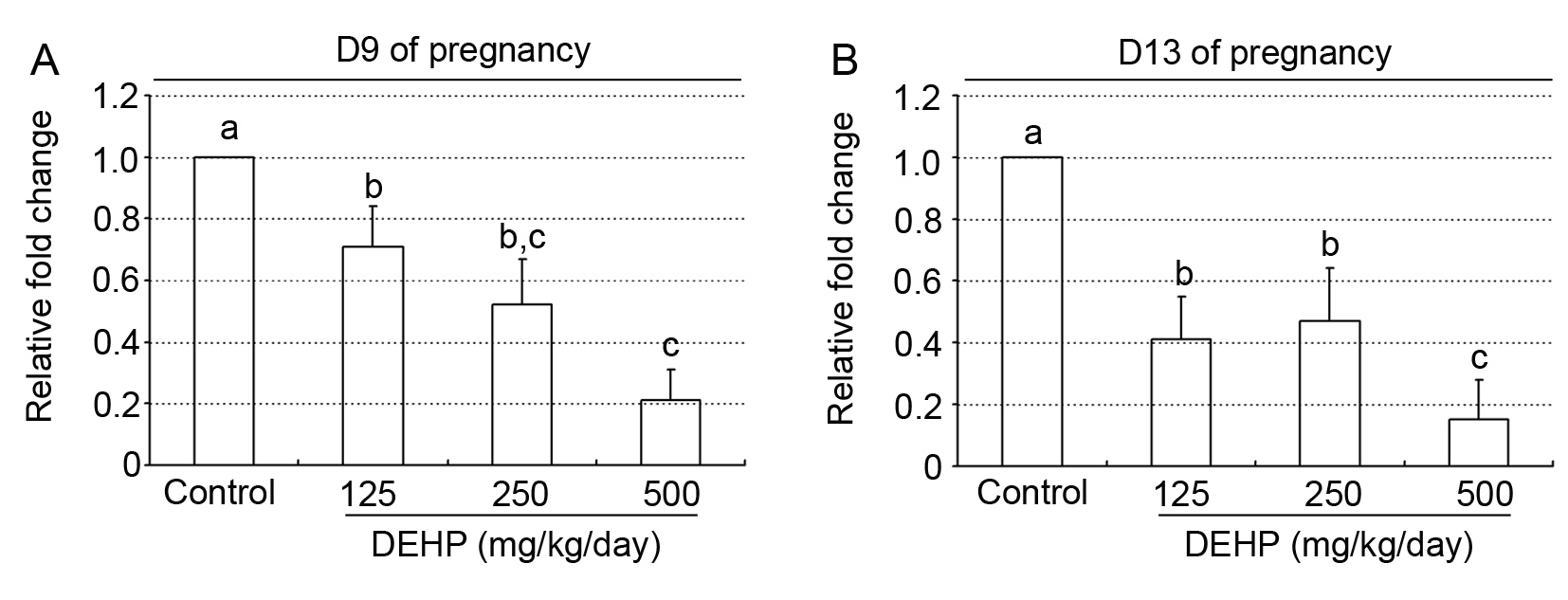

Supplement: Additional file 1: — Relative expression levels of CD31 protein detected by immunohistochemistry. (A, B) Relative expression levels of CD31 protein in the ovaries on day 9 (A) and 13 (B) of pregnancy. Groups with different superscript letters are significantly different (P < 0.05, ANOVA followed by LSD multiple range test). [file 12958_2015_13_MOESM1_ESM.jpeg]

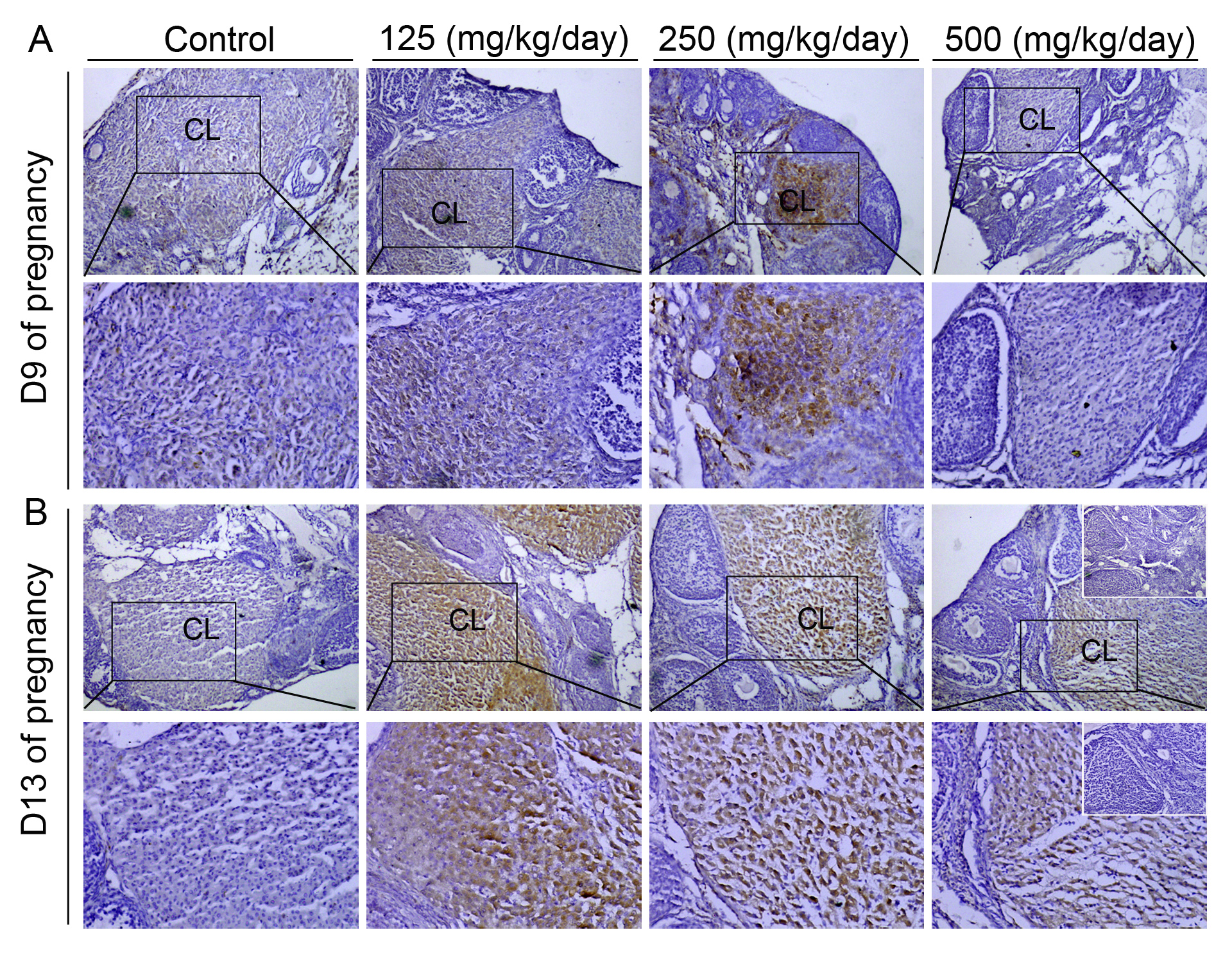

Supplement: Additional file 2: — The expression of COX2 protein detected by immunohistochemistry. (A, B) Representative images of ovaries on day 9 (A) and 13 (B) of pregnancy. Squared areas at the top (×100) are presented at higher magnification (×200) at the bottom. Inset is negative control. CL, corpus luteum. [file 12958_2015_13_MOESM2_ESM.jpeg]

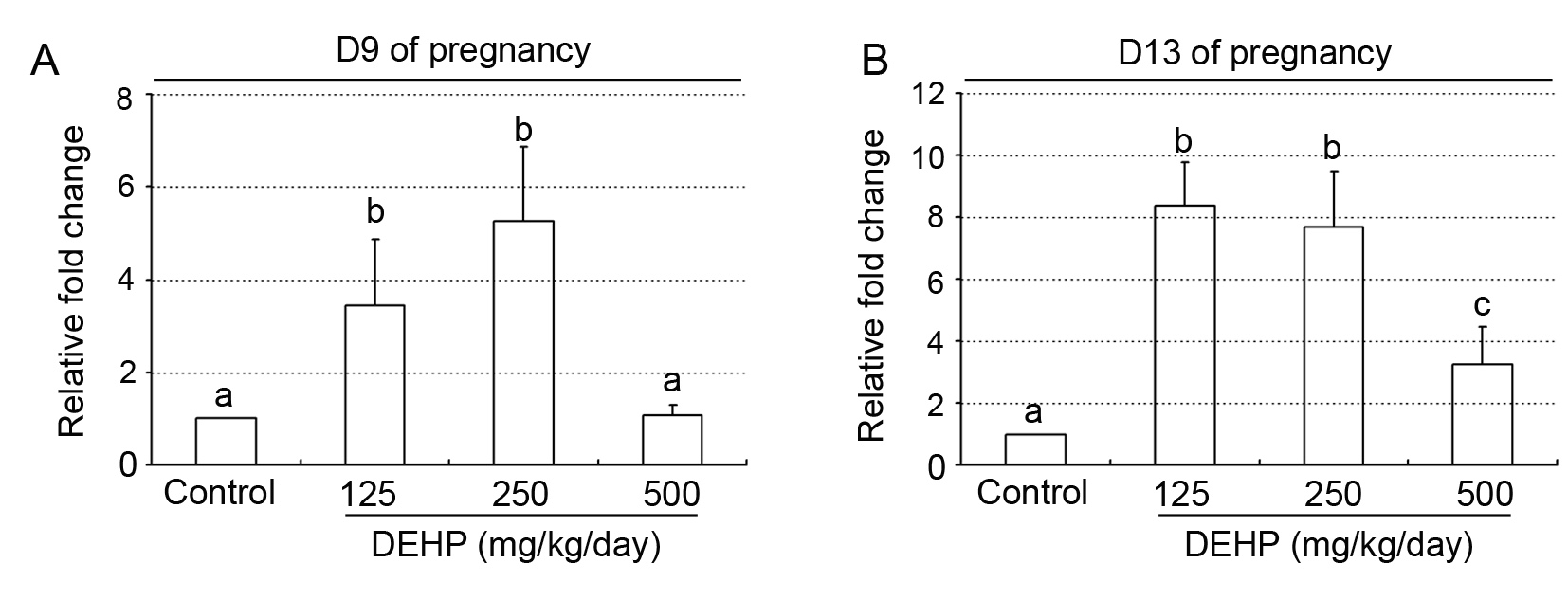

Supplement: Additional file 3: — Relative expression levels of COX2 protein detected by immunohistochemistry. (A, B) Relative expression levels of COX2 protein in the ovaries on day 9 (A) and 13 (B) of pregnancy. Groups with different superscript letters are significantly different (P < 0.05, ANOVA followed by LSD multiple range test). [file 12958_2015_13_MOESM3_ESM.jpeg]
